# Microbiota and Immune-Mediated Skin Diseases—An Overview

**DOI:** 10.3390/microorganisms7090279

**Published:** 2019-08-21

**Authors:** Adrian Catinean, Maria Adriana Neag, Andrei Otto Mitre, Corina Ioana Bocsan, Anca Dana Buzoianu

**Affiliations:** 1 Department of Internal Medicine, Iuliu Hatieganu University of Medicine and Pharmacy, 400337 Cluj-Napoca, Romania; 2 Pharmacology, Toxicology and Clinical Pharmacology Department, Iuliu Hatieganu University of Medicine and Pharmacy, 400337 Cluj-Napoca, Romania; 3 Faculty of Medicine, Iuliu Hatieganu University of Medicine and Pharmacy, 400337 Cluj-Napoca, Romania

**Keywords:** microbiota, immune-mediated diseases, autoimmune skin diseases, probiotics

## Abstract

In recent years, increased attention has been paid to the relationship between microbiota and various diseases, especially immune-mediated diseases. Because conventional therapy for many autoimmune diseases is limited both in efficacy and safety, there is an increased interest in identifying nutraceuticals, particularly probiotics, able to modulate the microbiota and ameliorate these diseases. In this review, we analyzed the research focused on the role of gut microbiota and skin in immunity, their role in immune-mediated skin diseases (IMSDs), and the beneficial effect of probiotics in patients with this pathology. We selected articles published between 2009 and 2019 in PubMed and ScienceDirect that provided information regarding microbiota, IMSDs and the role of probiotics in these diseases. We included results from different types of studies including observational and interventional clinical trials or in vivo and in vitro experimental studies. Our results showed that probiotics have a beneficial effect in changing the microbiota of patients with IMSDs; they also influence disease progression. Further studies are needed to better understand the impact of new therapies on intestinal microbiota. It is also important to determine whether the microbiota of patients with autoimmune diseases can be manipulated in order to restore homeostasis of the microbiota.

## 1. Introduction

Antibiotic therapy is one of the most effective forms of therapy known to man. The 1940s was called the “golden age of antibiotics” because it followed the discovery of penicillin by Fleming et al. and the production of penicillin on an industrial scale [1].

For this reason, most researchers have focused on identifying pathogenic bacteria and developing antimicrobial substances against these species. Currently, antibiotics are recognized for their utility in treating and preventing bacterial infections. However, they have a negative impact on the commensal (good) bacteria of the body.

Since 1950, interest in identifying nutraceuticals to inhibit the excessive growth of pathogens has increased. Probiotics (bios or “for life”) are defined as viable species of microorganisms that, when administered, modulate gastrointestinal flora and provide health benefits [2,3,4].

Gastrointestinal flora is a part of the human microbiota consisting of trillions of microbes living on and within humans. Microbiota include the total microorganisms that share our body space and colonize different areas of the body such as the skin, nasal cavities, oral cavities, eyes, and the genitourinary tract [5,6]. The microbiome, sometimes called the second genome, is formed by a large and diverse community of microorganisms (bacteria, fungi, phage, and viruses) and their genes [7]. The gut microbiota, also known as the intestinal microbiota or intestinal flora, consists of all bacteria, viruses, or other microorganisms that colonize the gastrointestinal tract [8]. At this level, the main essential (beneficial) and opportunistic (pathogenic) bacteria are: *Lactobacillus* spp., *Enterococci*, and *Propionibacterium* (essential); and *Bacteroides*, *Clostridia*, *Enterobacteria*, *Actinobacteria* and *Staphylococci* (opportunistic) [9]. *Bacteroides*, *Firmicutes*, *Proteobacteria*, and *Actinobacteria* are the main bacterial phyla identified in the fecal microbiota of healthy individuals [5].

The skin is the most exposed organ of the body in regard to environmental changes and stress. It possesses a dynamic and complex microbial ecosystem [10]. The bacteria that live on the skin are closely related in type and density to those found in skin glands or hair follicles in different areas. For example, *Staphylococcus* is a dominant species in both the sebaceous and moist areas, while *Propionibacterium* dominates only in the sebaceous and *Corynebacterium* only in the moist areas [11].

**Figure 1:**
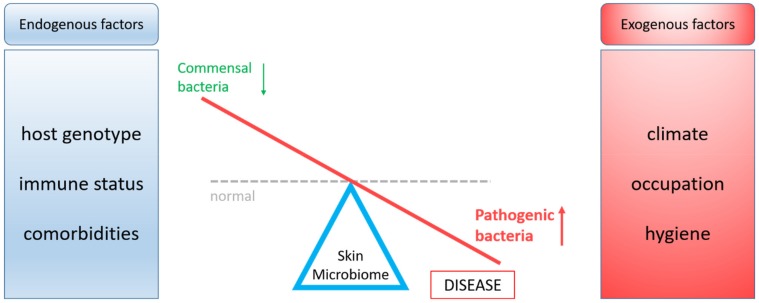
Impact of endogenous and exogenous factors on the skin microbiome dashed line, normal (balanced) microbiota; red line, microbiota in pathologies

In healthy individuals, there is a balance between essential and opportunistic bacteria, while in a pathological state dysbiosis occurs. The balance between beneficial and pathogenic skin bacteria can be disturbed by endogenous or exogenous factors (Figure 1) [11]. The current modern lifestyle habits (e.g., diet, stress, and sedentariness) can change the microbiota composition and may lead to disturbances of immune system homeostasis [12,13,14].

The available body of evidence has shown links between intestinal microbiota and autoimmune diseases (arthritis, psoriasis, diabetes, and others) that target different tissues (joints, skin, and others), not only in those that target the intestine [15,16,17,18].

This review aims to analyze recent information supporting an association between the gut and skin microbiota composition, immune-mediated skin diseases (IMSDs), and the beneficial effect of probiotics in these pathologies.

## 2. Survey Methodology

We performed an electronic literature search in the PubMed and ScienceDirect databases; relevant articles published between 2009 and 2019 were included. We used the following search terms: “nutraceuticals”, “probiotics”, and “health benefits” in combination with “skin microbiota”, “gut microbiota”, “gastrointestinal bacteria”, “microbiome”, “immune mediated diseases”, and “autoimmune skin diseases”. In this review, we included evidence from various types of studies including interventional, observational, and experimental studies and covering both in vitro and in vivo research.

## 3. Skin Microbiota and Immunity

In recent years, IMSDs have become a major public health problem [19,20]. Like other autoimmune diseases, IMSDs are caused by an inappropriate activation of the immune system [21]. Skin-resident microbes have the ability to modulate skin immune homeostasis and are therefore potentially part of the mechanism behind IMSDs [22].

The outer layers of the skin consist of the epidermis and dermis; a large percentage of the epidermis (up to 95%) consists of keratinocytes [23]. These cells have an extraordinary ability to divide and can regenerate the epidermis through self-renewal. Healthy keratinocytes function to provide a physical and chemical barrier against pathogens; they can also control the immune response of the skin [24]. Keratinocytes possess pattern recognition receptors (PRRs), by which they interact with microbial lipoproteins, nucleic acids, and cell wall components. Activation of PRRs increases the expression of antimicrobial peptides, cytokines, and chemokines [25]. Keratinocytes can also produce antimicrobial proteins (AMPs) that inhibit the growth of, or even destroy, various pathogenic bacteria. AMPs can influence cell membrane permeability and may act as bactericidal agents via an intracellular pathway [26].

Many AMPs play roles in immune reactions (Table 1). Some are constitutively expressed in the skin and, in the case of disruption to the microbiota, their expression can be upregulated by certain bacterial species (such as *S. epidermidis* or *Propionibacterium* spp.). In addition, skin expression of AMPs can be regulated by the C5a complement receptor. The ultimate goal of AMP upregulation is to eliminate pathogenic microbial species such as *S. aureus* [27].

Although AMPs are best known as important mediators of the innate immune system, they have several functions in addition to the antimicrobial effect including cell proliferation, cell differentiation, and wound healing [49]. Moreover, AMPs stimulate the production of cytokines and chemokines.

Langerhans cells (LCs) are another type of cell with an important role in the immune function of the skin [24]. LCs are dendritic cells (antigen presenting cells) of the skin, located at the interface of the skin and the environment [50]. This location suggests the primary role of LCs is immune mediation in the skin barrier [51].

Keratinocytes and LCs present toll-like receptors (TLRs). These receptors can recognize various microbial pathogens and initiate an immune response. To better understand the pathophysiology of IMSDs, it is important to distinguish between innate immunity and adaptive immunity and to understand the roles of each type of immunity in disease.

The immune system of the skin is characterized by the ability of different cells (innate immune cells: macrophages, dendritic cells, natural killer cells) to communicate with epithelial cells and together trigger a specific immune response [52]. Although epithelial cells are not considered innate immune cells, epithelial cells in the intestine express several types of innate immune receptors. Maintenance of intestinal homeostasis depends on the expression of these receptors and the transduction of active signals on the microbiota [53].

## 4. Gut Microbiota and Immunity

Current thought is that the gut microbiota represents an important gateway to understanding the physiopathology and mechanism of many diseases [54]. Microbiota can establish relationships with the host, these interactions result in modulation of host immunity and hence influence many physiological functions [55]. Intestinal bacteria play an important role in modulating T cell function (T helper [Th]1, Th2, Th17) [56]. Innate and adaptive immunity structures form a complex network with an important role in adapting and responding to various external and internal environmental challenges [57]. An immune ‘firewall’ of mucus membranes is located in the gut. This ‘barrier’ consists of epithelial cells, mucus, antimicrobial proteins, immune cells, etc. [58].

Goblet cells of the gastrointestinal track constitutively secrete mucus and play an important role in reducing the exposure of luminal antigens to the immune system cells [59].

Paneth cells, also located in the intestine, secrete a viscous fluid containing lysozyme, mucin 2 and antibacterial peptides. From the antibacterial peptides, α-defensins—together with IgA, neutrophils, and the innate lymphoid cells—form a strong defensive line against pathogens [60].

Because the microbiota continuously stimulates immune reactivity within the host, it is a key element for developing a strong immune system. In addition, an imbalance of homeostasis within the microbiota, especially after birth and in a child’s early years, causes important changes in the maturation and later function of the immune system. Any disturbance can predispose the individual to immune and/or inflammatory pathology later in life [61].

**Figure 2:**
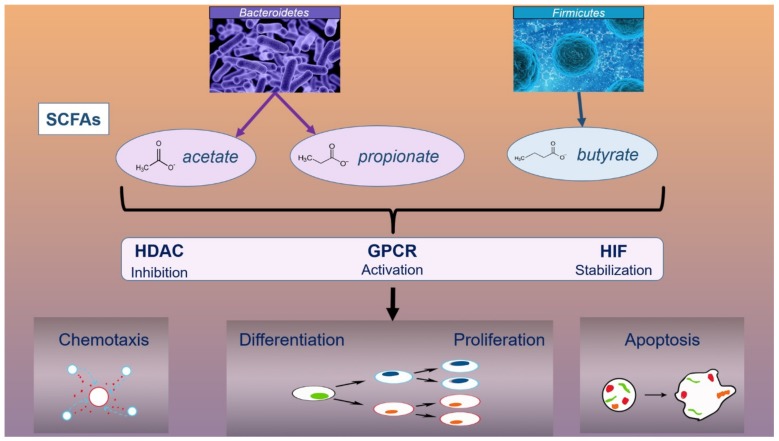
The role of SCFAs in immunomodulation Abbreviations: SCFAs, short chain fatty acids; HDAC, histone deacetylases; GPCR, G protein coupled receptors; HIF, hypoxia-inducible factor.

Short chain fatty acids (SCFAs) have an immunomodulatory effect which can be explained by cellular processes in which SFCAs are involved. These cellular processes include chemotaxis, differentiation, proliferation, and apoptosis, all of which occur as a result of the influence of SFCAs on several signaling pathways: inhibition of histone deacetylases (HDAC), activation of G protein coupled receptors (GPCR), and stabilization of the hypoxia-inducible factor (HIF) (Figure 2) [62,63]. SCFAs inhibit the secretion of inflammatory cytokines (TNF-α, IL-6, IL-17, IFN-γ), likely by lipopolysaccharide (LPS)-induced chemokines and cytokines, and modulate the ratio between good and pathogenic bacteria by suppressing the overgrowth of pathogens [64,65].

Acetate, propionate, and butyrate are the main SCFAs and the main nutrients produced by fermenting dietetic components (carbohydrates) under the action of gut bacteria [66]. Acetate and propionate are produced by microbial species such as *Bacteroidetes*, while butyrate is produced by members of the *Firmicutes* family (*Lachnospiraceae, Ruminococcaceae*) [67,68]. Although butyrate is the main SCFA, it is produced by only a few bacteria in the intestine: *Faecalibacterium prausnitzii, Eubacterium rectale, Eubacterium hallii*, and *Ruminococcus bromii* [69].

HDAC inhibitors are currently being explored as therapeutic options to restore the immune response of the body and to inhibit excessive immune responses in autoimmune diseases [70].

All SCFAs inhibit HDAC activity and suppress the activity of regulatory T cells (Treg) cells that play a key role in immunological homeostasis [71].

Some of the most common IMSDs are atopic dermatitis, psoriasis, vitiligo, scleroderma, pemphigus vulgaris, bullous pemphigoid, lichen planus, erythema multiform, and systemic lupus erythematosus. The main changes of skin and gut microbiota in patients with IMSDs are presented in Table 2.

## 5. Role of Microbiota in IMSDs

### 5.1. Atopic Dermatitis

Atopic dermatitis (AD) is one of the most common immune-mediated skin diseases and has a significant impact on pediatric health. The pathophysiology involves both the disruption of the skin’s epithelial barrier and an abnormal immune response [93].

*S. aureus* is one of the most studied microbial agents of the skin. It is considered commensal in approximately 30% of the human population and is mainly located in the nose. The prevalence of *S. aureus* is increased in patients with AD, especially within lesional skin. This was shown in a meta-analysis, which included 91 observational studies in AD patients, where colonization with *S. aureus* was greater in lesional (70%) compared with non-lesional skin (39%) or nasal mucosa (62%) [94,95].

The microbial diversity of the skin is reduced in patients with AD. Thus, *S. aureus* is able to proliferate on the skin and acts to modify the ratio of Th1/Th2 cells [96]. This modification leads to secretion of Th2 cytokines (IL-4, IL-5, IL-13) and IgE as well as the stimulation of *S. aureus* binding on the affected skin [97].

*S. aureus* is not the only factor involved in the pathophysiology of AD. There is a complex interaction between factors from the host and pathogen; on the one hand, host factors provide chemical, physical, and antimicrobial properties to the skin, while on the other hand, pathogens possess mechanisms that interfere with adhesion and induce inflammation and immunological changes [90].

Song et al. demonstrated a reduction of *Faecalibacterium prausnitzii* spp. in patients with AD compared with healthy population [98]. Dysbiosis contributes to disruption of the intestinal barrier integrity and increased permeability that allows microbes and toxins to enter into the systemic circulation and to reach target tissues, including the skin. It is known that *Faecalibacterium prausnitzii* spp. stimulate the SCFA production in the gut. These acids have an anti-inflammatory and beneficial role for the intestinal health [99,100].

AD patients present a complex skin immune response. In the acute stage of the disease, keratinocytes release the cytokines TSLP (tymic stroma lymphopoetin), IL-25 and IL-33 that will stimulate the Th2 immune response. In chronic stages Th-22 cells release IL-22 and stimulate the production of AMP (e.g., defensins) from the epiderma. This reaction modifies the immunity towards a more Th1 predominant response [100].

We must also take into account genetic factors in AD. It has been demonstrated that filament aggregating protein (filaggrin) and null mutations within genes encoding it (FLG genes) are closely associated with the predisposition of individuals to develop AD [101,102]. Filaggrin is also an important factor involved in the differentiation of keratinocytes [103].

Other studies observed a reduction in *Streptococcus*, *Corynebacterium*, and *Propionibacterium* spp. during AD flares. This change in abundance of bacterial species on the skin could be a direct consequence of the action of antimicrobial compounds secreted by *S. aureus* and *S. epidermidis* [92]. Similar dysbiosis of the skin microbiota is caused by pharmaceuticals used to treat AD [104].

The objectives of AD treatment are restoring the skin barrier, reducing skin inflammation, managing triggers (stress, allergy, etc.), and restoring the normal skin microbiome or treating microbial infections [93].

### 5.2. Psoriasis

Psoriasis is a chronic inflammatory skin disease that affects 2–4% of the world’s population [105]. Several factors such as genetics and factors that cause disruption of the skin barrier and immune dysfunction, are involved in the onset and progression of this disease [106].

Over the past 20 years, considerable progress has been made in understanding the pathogenesis and treatment of psoriasis [107]. Microorganisms such as bacteria (*S. aureus*, *S. pyogenes*), fungi (*Malassezia*, *Candida albicans*), or viruses (certain retroviruses) are involved in psoriasis pathogenesis [108,109]. In the blood of patients with plaque psoriasis (most common variation), a higher amount of bacterial DNA was found for some species of *E. coli*, *Klebsiella pneumoniae*, *Enterococcus faecalis*, *Proteus mirabilis*, and *S. pyogenes* [110]. Compared to healthy individuals, the stool of psoriasis patients consists of a lower abundance of *Faecalibacterium prausnitzii* and a greater abundance of *E. coli* [111]. Fahlen et al. showed that the level of *Actinobacteria* was significantly increased in skin from healthy individuals compared with that of psoriasis patients, while *Proteobacteria* dominated in lesional skin. Additionally, they observed a higher ratio of *Streptococcus*/*Proteobacteria* in the skin of these patients [112].

Codoner et al. found that psoriasis patients had decreased levels of *Bacteroides* spp. in the intestine compared to the healthy population. *Bacteroides* spp. interfere with the body’s ability to react to external pathogens. These bacteria activate Treg by stimulating polysaccharide A [79]. Some studies observed a direct link between bacterial DNA and cytokine levels (TNF-α, IFN-γ, IL-1b, IL-6, IL-12, IL-22) [113]. It is known that human immune cells respond to the presence of bacterial DNA in a Toll-like receptor 9 (TLR9)-dependent manner; hBD3, a β-defensin, has the ability to further stimulate this response [114].

Dysbiosis of the intestine favors the ‘leaky gut’ phenomena of increased intestinal permeability. This results in bacterial translocation and a chronic inflammatory state in the host [115].

The results of psoriasis and microbiota studies offer new perspectives on the treatment of this disease. Thus, antibiotics, probiotics, and prebiotics—through targeting of the microbiota and its homeostasis—could influence disease prognosis and progression.

### 5.3. Vitiligo

Vitiligo, another IMSD, is characterized by the appearance of depigmented macules on the skin [116]. This manifestation occurs because of the destruction of melanocytes in the skin by T cells, mononuclear cells, pro-inflammatory cytokines, and/or autoantibodies [117,118].

Several mediators are linked to the activities of melanocytes and include stem cell factor (SCF), basic fibroblast growth factor and endothelin-1 (ET-1) [119]. SCF is a promelanogenic factor that promotes melanocyte growth, differentiation, migration, and survival [120]. In patients with vitiligo, both SCF and ET-1 are reduced in lesional skin compared to non-lesional skin [121]. ET-1 released from epidermal keratinocytes under the action of stress factors (e.g., narrow band B ultraviolet radiation) also contributes to the proliferation, differentiation and migration of keratinocytes [122]. In addition to these mediators, other cytokines secreted by dermal fibroblasts (IL-1α, TNF-α) influence the function and survival of melanocytes and also stimulate SCF production [123].

A study reported by Ganju et al. analyzed the cutaneous microbiota in patients with vitiligo. They observed a reduction of microbiota diversity in lesional sites compared with non-lesional sites; *Actinobacteria* represented the dominant taxa in non-lesional skin while *Firmicutes* and *Proteobacteria* dominated in the lesional sites [83].

### 5.4. Systemic Lupus Erythematosus

Systemic lupus erythematosus (SLE), an autoimmune disease with a higher prevalence in women than men, is characterized by overactive immune cells and abnormal antibody responses to cellular antigens [124]. Although the exact etiology of SLE is unknown, one hypothesis is that the disease is a consequence of a complex relationship between genetic and environmental factors [125].

Another hypothesis states that the microbial composition of the intestine and oral cavity may influence the etiopathology of SLE. This idea is supported in a study by van der Meulen et al. that compares the oral and intestinal microbiota of patients with SLE with that of the healthy subjects. The results demonstrate a higher abundance of *Bacteroides thetaiotaomicron* (*B. theta*) (a potential intestinal pathobiont) in patients with SLE compared to the control group.

Regarding the microbiota of the oral cavity, its composition is coordinated by diseases-related changes at this level [126]. In patients with SLE, one of the most studied dysbiosis-changes is the *Firmicutes/Bacteroidetes* ratio. This is lower in patients with SLE compared to the healthy population.

Only a few studies have shown the presence of dysbiosis in patients with SLE. Specifically, a lower ratio of *Firmicutes*/*Bacteroides* was identified in SLE patients in both Spain and southern China, the increase in *Actinobacteria* was only significant in patients from the latter region [124,127].

Changes in the gut microbiota in SLE patients (for example, increases in *Veillonella* or *Fusobacterium* spp., along with decreases in *Bacteroides uniformis*) are strongly linked to "leaky-gut" syndrome, which causes inflammatory and abnormal immunity conditions [128]. Changes in the composition of the gut microbiota are correlated with the clinical course of the disease. Serum levels of IFN-γ, which are slightly reduced in SLE, are reported to be directly related to the amount of *Firmicutes* and the *Firmicutes*/*Bacteroides* ratio in these patients [129].

## 6. Probiotics–Microbiota Targeted Treatment in IMSDs

Even though IMSDs are becoming more common and have a negative impact on patient’s lives, treatment options or prophylaxis are limited and the identification of chemically beneficial compounds in IMSDs is costly. Therefore, studies investigating the role of nutraceuticals in these pathologies have been a major focus of research in this field over the last decade.

Our current understanding of the role of microbiota in the pathogenesis of autoimmune diseases suggests that microbiome manipulation could pose as a promising solution for promoting the remission of disease and restoring homeostasis.

Probiotics improve immunity through both indirect and direct actions, but the exact mechanisms are not yet fully understood. Indirect effects may be exerted either by increasing the production of SCFAs or AMPs or by restoring the gut epithelial barrier; direct effects are a consequence of the interactions between probiotics and innate immune receptors, thus influencing signaling pathways (NF-κB, MAPK) and decreasing proinflammatory cytokines [130].

A nutraceutical product consisting of *Lactobacillus* spp. (*L. casei, L. rhamnosus*, *L. plantarum*), *Bacillus lactis*, fructooligosaccharide, galactooligosaccharide, and biotin was studied in 275 children with AD. This study demonstrated that the symbiotic supplement ameliorated this autoimmune skin disease and reported good safety and tolerability results [131].

However, not all studies have shown a beneficial effect of probiotics. For example, Brouwer et al. have demonstrated that probiotics containing *L. rhamnosus* or *Lactobacillus* GG have neither clinical nor immunological effects in infants with AD [132]. These conflicting findings may depend on several factors including patient age (infants, children, or adults), type of delivery (caesarean or vaginal delivery), early infant feeding (breastfeeding or non-breastfeeding), drug history (e.g., antibiotics) or other factors; all factors listed influence the human microbiome [133,134,135].

In other studies, the effect of probiotics on psoriasis was evaluated. *Bifidobacterium infantis* was administered to patients with psoriasis for 2 months; plasma levels of C-reactive proteins and the proinflammatory cytokines TNF-α and IL-6 were determined. The results showed that probiotic treatment significantly decreased all proinflammatory parameters [136].

## 7. Conclusions

Microbiota, large and dynamic populations of microorganisms that lives with us from our first days of life, have been neglected for a very long time. In recent years, many studies have analyzed the relationship between microbiota and hosts in both healthy and sick individuals, resulting in an understanding that the microbiota balance and human health are closely related.

A better understanding of the microbiota, especially of gut and skin, is undoubtedly useful in understanding the main aspects of the etiology and pathophysiology of IMSDs. This understanding is useful for developing new therapies with improved efficacy and safety.

Additional studies are needed to better understand the impact of new therapies on the intestinal microbiota. It is also important to determine whether the microbiota of patients with autoimmune diseases can be manipulated in order to restore homeostasis to the microbiota and alleviate such diseases.

## Figures and Tables

**Table 1:** Link between antimicrobial peptides and effects on microbiota

AMP	Types	Localization	Effect	Ref.
Cathelicidins	hCAP-18 (LL-37)	Neutrophils Macrophages Skin epithelial cells Bone marrow Gastrointestinal tract Lungs	Antibacterial activity (against Gram-positive and Gram-negative strains) (*Acinetobacter baumannii*, *E. coli*, *Fusobacterium nucleatum*, *Haemophilus influenza*, *Salmonella* spp., *Propionibacterium acnes*, *S. aureus*, etc.) Effector molecules of the innate immune system	[28,29,30,31]
Defensins	α-Defensins 1–4 (human neutrophils peptides [HNP]) α-defensins 5-6 (HD-5, HD-6)	NK cells Monocytes T lymphocytes Paneth cells of the small intestine HD-5 in the genital tracts	HNP-1: induces TNF-α expression HNPs-1–3: chemoattractant effect for monocytes, immature DCs, naïve CD4+ T cells HNP-1 and HNP-3: induce the migration of macrophages and mast cells HD-5: bactericidal effect (against Gram-positive strains) HD-5: stimulates the production of IL-8	[32,33,34]
	β-Defensins	Epithelial cells hBD-1, hBD-2 in salivary gland	Antibacterial activity (against Gram-positive and Gram-negative strains) chemoattraction of macrophages, monocytes, T cells, mast cells, neutrophils, immature DCs, and fibroblasts	[35,36,37,38,39]
Ribonuclease	RNase 5 RNase 7	Epidermis Liver Gastrointestinal tract Kidney Heart Skeletal muscle Respiratory tract	Bactericidal activity (against Gram-positive and Gram-negative strains) (*Streptococcus pneumoniae*, *Listeria monocytogenes*, *E. coli*, *Pseudomonas aeruginosa*, *S. aureus*, *Enterococcus faecium*, *Propionibacterium acnes*)	[40,41,42]
Dermicin	DCD-1L DCD-1	Sweat glands	Antimicrobial activity (against Gram-positive and Gram-negative strains) (*S. aureus*, *E. faecalis*, *S. epidermidis*, *Listeria monocytogenes*, *E. coli*, *Pseudomonas putida*, *Salmonella typhimurium*)	[43,44]
Psoriasin (S100A7)		Keratinocytes of healthy humans Epithelial cells of patients with inflammatory or malignant diseases of the skin, breast, bladder urinary tract, otolaryngology zone	Antimicrobial activity against *E. coli* (lower doses), *P. aeruginosa* (higher doses)	[45,46,47,48]

Abbreviations: hCAP-18, human cathelicidin; LL-37, human cathelicidin LL-37; HNP, human neutrophil peptides; NK, natural killer; TNF, tumor necrosis factor; DC, dendritic cell; CD, cluster of differentiation; HD, α-defensins; hBD, β-defensins; RNase, ribonuclease; DCD, dermicin.

**Table 2:** The link between several autoimmune skin diseases and changes of microbiota.

Disease	Changes in the Diversity and Composition of the Microbiota	Consequences	References
**Scleroderma (systemic sclerosis)**	**Gut dysbiosis:** ↓ *Faecalibacterium* ↓ *Clostridium* ↓ *Bacteroides* ↑ *Fusobacterium* ↑ *Prevotella*	*Faecalibacterium:* produces butyrate (involved in SCFA production), associated with anti-inflammatory activities (downregulation of NF-kB activation and IL-12, IFN-γ and IL-8 production) *Clostridium:* produces exotoxins which lead to IBS and pseudomembranous colitis *Bacteroides:* capsule can form abscesses *Fusobacterium:* associated with gastric cancer, may be involved in oncogenesis *Prevotella*: promotes inflammatory responses by stimulating IL-8, IL-6, and CCL-20 production and Th17 mediated immune responses	[72,73,74,75]
**Psoriasis**	No definitive association: **Lesional skin:** *↑ S. pyogenes* *↑S. aureus* *↓Malassezia* *↓Cutibacterium* Inconclusive: **Gut**: *↓/↑ Akkermansia muciniphila* *↓/↑ Ruminococcus*	*S. pyogenes*: promotes chronic skin inflammation through SPE-C secretion and via streptococcal antigens in the dermal layers *S. aureus*: via staphylococcal α-toxin released in the skin and secretion of superantigens *Malassezia* and *Cutibacterium*: involved in immunomodulation and the skin protective barrier; decrease can aggravate disease *Akkermansia* and *Ruminococcus*: mucolytic agents (transforming mucin in SCFAs), associated with the intestinal barrier function	[76,77,78,79,80]
**Bullous pemphigoid**	**Perilesional skin:** ↑ *Firmicutes* ↑ *S.* *epidermidis* ↓ *Actinobacter*	No clear evidence on how dysbiosis can causes the disease; changes in perilesional microbiota are likely a consequence of the pathology *Firmicutes* and *Actinobacter:* involved in carbohydrate metabolism and produce SCFAs (including butyrate) *S. epidermidis:* promotes immune response via IL-17A and IFN-γ	[81,82]
**Vitiligo**	**Lesion skin:** ↑ *Firmicutes* ↓ *Actinobacter* ↓ *Corynebacterium* **Gut microbiota:** Dysbiosis of the gut microbiota after ampicillin and neomycin administration exacerbates disease	*Firmicutes* and *Actinobacter:* involved in carbohydrate metabolism and produce SCFAs (including butyrate) *Corynebacterium:* promotes inflammation of the skin via γδ T cell activation	[82,83,84,85]
**Atopic Dermatitis**	**Skin microbiota:** ↑ *S. aureus* ↑ *S. epidermidis* **Gut microbiota:** ↑ *Clostridium difficile* ↑ *S. aureus* ↓ *Bifidobacterium*	*S. aureus* is a main pathogen in AD, it is associated with disease onset and severity *S. aureus:* produces superantigen molecules, δ- and α- toxins, cytolysin, and MSCRAMMs *S. epidermidis:* promotes immune responses via IL-17A and IFN-γ *Clostridium difficile:* produces exotoxins, leading to IBS and pseudomembranous colitis *Bifidobacterium:* role in carbohydrate metabolism	[27,80,86,87,88,89,90,91,92]

Abbreviations: AD, atopic dermatitis; CCL, chemokine ligand; IFN, interferon; IL, interleukin; IBS, irritable bowel syndrome; MSCRAMMs, microbial surface components recognizing adhesive matrix molecules; NF-κB, nuclear factor kappa-light-chain-enhancer of activated B cells; SCFA, short chain fatty acid; SPE-C, superantigen streptococcal pyogenic exotoxin C.
